# FCGBP Promotes Glioma Growth by Regulating JAK2/STAT3/c‐Myc Pathway

**DOI:** 10.1002/cam4.71617

**Published:** 2026-02-15

**Authors:** Jin Zheng, Yu xin Rao, Hui Zheng, Liang liang Shi

**Affiliations:** ^1^ Department of Neurosurgery Union Hospital, Tongji Medical College, Huazhong University of Science and Technology Wuhan Hubei China; ^2^ Department of Radiology Union Hospital, Tongji Medical College, Huazhong University of Science and Technology Wuhan Hubei China; ^3^ Cancer Center Union Hospital, Tongji Medical College, Huazhong University of Science and Technology Wuhan Hubei China

**Keywords:** FCGBP, glioma, JAK2/STAT3/c‐Myc, progression

## Abstract

**Background:**

FCGBP has been implicated in the development of a variety of tumors, but its exact role in glioma progression remains unclear.

**Methods:**

This study utilized qRT‐PCR, Western blotting (WB), immunohistochemistry (IHC), and other techniques to assess FCGBP expression in glioma tissues. Additionally, various functional experiments were conducted to investigate its role in glioma progression. Animal models were employed to further elucidate the function of FCGBP, with a particular focus on its impact on the Janus Kinase 2/Signal Transducer and Activator of Transcription 3 (JAK2/STAT3) signaling pathway.

**Results:**

The research findings indicated that FCGBP is significantly overexpressed in glioma and is closely associated with higher tumor grades and unfavorable clinical outcomes. Functional assays demonstrated that FCGBP promotes glioma cell aggressiveness by activating the JAK2/STAT3 signaling pathway.

**Conclusion:**

This study highlights the critical role of FCGBP in glioma aggression and poor prognosis, indicating that it could serve as a potential therapeutic target.

## Graphical abstract

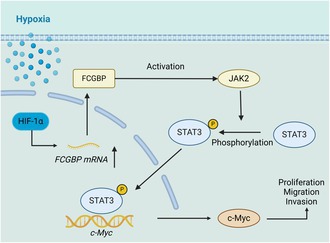

Schematic representation of the proposed mechanism. FCGBP is highly expressed in gliomas and is negatively correlated with patient prognosis, contributing to glioma progression. Mechanistically, FCGBP promotes glioma progression through two key pathways: (1) FCGBP activates the JAK2‐STAT3 signaling pathway, leading to the upregulation of c‐Myc protein levels; (2) In the tumor’s hypoxic microenvironment, HIF‐1α directly binds to the FCGBP promoter, enhancing FCGBP protein expression.

## 1 Introduction

Gliomas (GMs) represent the most prevalent central nervous system (CNS) primary malignancy, responsible for nearly 30% of all brain tumors and 80% of all brain malignancies. Although GMs can develop at any age, they are more frequent in adults, particularly among middle‐aged and elderly populations aged 50–70. The incidence rate in men is slightly higher than in women. Generally, GMs have a poor prognosis, especially glioblastoma (GBM) [1, 2]. The median survival of GBM patients is approximately 12–15 months, with a 5‐year survival rate of less than 5% [3, 4]. Given its escalated invasiveness, frequent recurrence, and poor prognosis, GM remains a major focus of neuro‐oncology research.

The IgG Fc‐binding protein (FCGBP) is a cysteine‐rich, mucin‐like protein that is widely expressed in the intestine, lungs, and tumor tissues, playing a crucial role in mucosal barrier function, immune regulation, and cancer progression [5, 6]. In recent years, the role of FCGBP in malignant tumors such as GMs and colorectal cancer (CRC) has gained increasing attention [7, 8]. However, the role of FCGBP in GM pathogenesis remains unexplored, with no studies to date specifically investigating its function in this context. According to the results from GSEA analysis, FCGBP is most closely related to the JAK/STAT signaling pathway. We further investigated the response of the JAK/STAT pathway to FCGBP using Western blotting and found that the p‐JAK2/p‐STAT3 response was the highest. Interestingly, Lu et al. reported that FCGBP may promote ovarian cancer progression by regulating the JAK2‐STAT3 signaling pathway. Therefore, we also hypothesized that FCGBP interacts with the JAK2/STAT3 pathway to promote glioma progression.

The key pathway, Janus Kinase 2/Signal Transducer and Activator of Transcription 3 (JAK2‐STAT3), plays a crucial role in contributing to cell proliferation, differentiation, survival, and immune regulation [9, 10]. It is highly activated in GMs, particularly in GBM, and contributes to tumor progression, treatment resistance, and immune evasion [11]. However, whether FCGBP plays a role in mediating this activation remains vague.

Therefore, we aimed to explore the FCGBP function in GM, uncover novel mechanisms that drive GM progression, and identify potential therapeutic targets for future treatment strategies.

## 2 Materials and Methods

### 2.1 Cell Lines and Reagents

Before the study, STR DNA fingerprinting was performed to authenticate cell lines obtained from the Chinese National Infrastructure of Cell Line Resource (NHA, H4, U‐251, T98G, LN‐18) and the American Type Culture Collection (HA, U118MG, A‐172, LN‐229, U‐87MG). The used cell lines were authenticated by an expert before use in experimentation and were free from mycoplasma contamination. They were maintained at 37°C in a 5% CO_2_ incubator and cultured in DMEM (Hyclone) that contained 10% FBS and 1% penicillin–streptomycin (Gibco). To ensure experimental integrity, mycoplasma contamination was screened using the LookOut Mycoplasma PCR Detection Kit (Sigma‐Aldrich).

Readers are encouraged to consult the published article for more details on the experimental methods [12, 13]. The compound WP1066 (S2796) was sourced from Selleck, China. Table S1 provides a comprehensive list of all the antibodies used.

### 2.2 Patients

To preserve the molecular integrity of tumor samples for future analysis, they were immediately cryopreserved at −80°C following neurosurgical GM resections. Before surgery, all patients provided informed consent, and their tissue samples were anonymized for processing, ensuring ethical compliance with ethical standards. Participants were explicitly informed through consent forms that their personal data would be kept confidential and that their tissue samples would be used for research purposes.

The collection and utilization of these samples were approved by the Human Research Committee of Wuhan Union Hospital and the China Anti‐Cancer Association. These regulatory bodies oversee the study to uphold ethical standards, safeguard patient rights, and ensure their welfare throughout the research process.

### 2.3 Plasmid Constructs and Transfection

Wuhan Genecreate Biological Engineering Co. Ltd. (Wuhan, China) provided lentiviruses carrying short hairpin RNAs (shRNAs) targeting FCGBP and c‐Myc, which were used for cell transduction and effectively suppressed the expression of FCGBP and c‐Myc. Table S2 lists the specific shRNA sequences employed for gene silencing, designed to degrade FCGBP and c‐Myc mRNA, thereby reducing their expression in transduced cells.

Control cells were infected with either an empty vector or scrambled shRNA to ensure a reliable comparison for assessing gene knockdown specificity and efficiency in FCGBP‐ and c‐Myc‐silenced cells. For an in‐depth description of the experimental methods and procedures, readers are encouraged to refer to previously published studies [14, 15].

### 2.4 Cell Migration and Invasion Assays

According to experimental requirements, we utilized a Transwell chamber having a suitably sized pore. For the invasion assay, Matrigel was applied to the chamber base to imitate the extracellular matrix. Pre‐treated tumor cells were suspended in a serum‐free medium and seeded into the upper chamber at a density of 5 × 10^3^ to 1 × 10^4^ cells/well density. The lower chamber contained a complete growth medium with 10% FBS to establish a chemotactic gradient, and the setup was incubated under optimal conditions.

After incubation, we carefully removed the non‐migrated cells on the upper membrane, and the chamber was rinsed with PBS to eliminate any remaining unattached cells. The membrane‐passed migrated/invaded cells that were adhered to the lower surface were fixed and stained with crystal violet, visualized, and counted under a microscope, providing a quantitative assessment of their migration and invasion capacity.

### 2.5 Luciferase Gene Reporter Assay

To construct the test plasmid, a dual‐luciferase reporter system was employed, with the target gene sequence inserted upstream of the luciferase reporter gene. A control plasmid containing an internal reference gene was co‐transfected to normalize the data. After culturing an appropriate cell line to the desired density, cells were then co‐transfected with both plasmids using a highly efficient transfection reagent, such as liposomes. To evaluate the target regulatory sequence activity, transfected cells were subjected to various experimental conditions.

Following treatment, cells were lysed to release luciferase proteins and luciferase activity was quantified using a luminescence detector. The data were normalized by calculating the ratio of target luciferase activity to internal reference activity, enabling a comparative analysis of relative luciferase expression levels under different experimental conditions.

### 2.6 Cell Counting Kit‐8 (CCK‐8) Assay

During the logarithmic growth phase, cells were resuspended and seeded into 96‐well plates at a density of 3800 cells/well. The initial culture medium was insufficient, lacking the essential components required for the experiment. After allowing 24 h for cell adhesion and monolayer formation, the medium was replaced, and 10 μL of CCK‐8 reagent was introduced to each well following the manufacturer's protocol.

The CCK‐8 assay evaluates cell viability by measuring metabolic activity. After the incubation period, the absorbance was measured at 450 nm using a microplate reader, with baseline readings subtracted to ensure accuracy in the final analysis.

### 2.7 Colony Formation Assay

To promote the formation of distinct colonies, we diluted and seeded the cells at 500 cells/well into 6‐well plates and cultured them at 37°C with 5% CO_2_ for approximately 2 weeks, until visible colonies formed. This process was repeated for various treatment groups. To maintain optimal conditions, the culture medium was regularly refreshed to supply nutrients and eliminate metabolic waste.

After incubation, the cells were fixed with 4% paraformaldehyde for 15–20 min, followed by crystal violet staining for 30 min to enhance colony visibility. Excess stain was carefully rinsed off, and colonies containing at least 50 cells were counted to calculate the colony formation rate, providing insight into the cells' proliferative capacity.

### 2.8 Bioinformatics Database

The TCGA database was accessed to retrieve gene expression data for the GM gene for analysis. Subsequently, cBioPortal and GraphPad Prism 10.1.2 (GraphPad Prism Software Inc., San Diego, California, USA) were utilized to conduct statistical and graphical analyses. For a comprehensive description of the methodologies and analytical procedures, readers are encouraged to refer to previously published articles [14, 16].

### 2.9 Western Blotting (WB)

Total protein from cell or tissue lysates was quantified using the BCA method to ensure consistency across samples. The proteins were then denatured by heat, mixed with loading buffer, and separated via SDS‐PAGE. Afterward, proteins were transferred onto nitrocellulose or PVDF membranes for subsequent immunodetection.

To minimize nonspecific binding, membranes were incubated at 4°C overnight with the primary antibody. After thorough washing, an HRP‐conjugated secondary antibody was applied to enhance signal detection, followed by a 2‐h incubation at room temperature. The target protein signals were visualized via an ECL chemiluminescent reagent, and band intensity was recorded using a chemiluminescent imaging system.

### 2.10 Immunofluorescence (IF) and Immunohistochemistry (IHC) Procedures

For IF staining, tissue sections were subjected to 4% paraformaldehyde fixation, 0.5% Triton X‐100 permeabilization, and a thorough wash with PBS. To minimize nonspecific binding, sections were treated with a 5% BSA blocking buffer. Subsequently, they were incubated overnight at 4°C with primary antibodies diluted in the same blocking buffer to specifically detect the target proteins. After primary antibody incubation, we introduced fluorescent dye‐conjugated secondary antibodies to bind the primary antibodies, generating fluorescence signals. Before mounting the sections with an anti‐fade solution, DAPI counterstaining was performed to visualize cell nuclei, ensuring fluorescence stability. High‐resolution imaging was conducted using laser scanning confocal microscopy, such as the Olympus FV500.

For IHC, 4‐μm‐thick formalin‐fixed, paraffin‐embedded tissue sections were prepared and incubated with a primary antibody targeting FCGBP, followed by a Cy3‐conjugated secondary antibody for detection. To quantify protein expression, the immunoreactive score (IRS) method was applied, calculated as the product of staining intensity (SI) and positive cell percentage (PP%). The SI was graded from 0 (negative) to 4 (very strong), while PP% was categorized from 0 (< 1%) to 4 (> 80%), yielding an IRS range of 0 to 16. Protein expression levels were assessed by evaluating 10 fields per sample. Table S1 lists the antibodies used.

### 2.11 Orthotopic GM Model in Nude Mice

Before tumor cell implantation, 6‐to‐8‐week‐old male/female nude mice were supplied by Beijing Vital River Laboratory Animal Technology Co. Ltd. and housed for 7 days in a specific pathogen‐free (SPF) environment to ensure acclimatization. Each experimental group consisted of three to five mice in independent trials. For tumor implantation, GM cells from distinct treatment groups were orthotopically injected into the mouse brain at a concentration of approximately 6 × 10^5^ cells per mouse. The injection site was accurately positioned 1 mm anterior to the bregma, 2 mm lateral to the right, and 3 mm in depth. A total of 5 μL of cell suspension was carefully injected at this location.

Two weeks post‐inoculation, mice were euthanized, and tumor volume was assessed using H&E and Ki‐67/TUNEL IF stainings to evaluate tumor proliferation and apoptosis.

### 2.12 Statistical Analysis

Statistical analyses were conducted through SPSS v25.0. For comparisons between two datasets, we applied unpaired and paired *t*‐tests, while using one‐way ANOVA for analyzing differences among more than two groups. The Shapiro–Wilk test was conducted to assess the normality of the data, with an alpha level of 0.05. Results are reported as mean ± SD or median with interquartile range (IQR). Based on normality and variance assumptions, we employed a standard *t*‐test, a variance‐corrected *t*‐test, or a rank‐sum test for two‐group comparisons. For multiple‐group comparisons, we utilized one‐way ANOVA, Brown‐Forsythe ANOVA, or a rank‐sum test. Pairwise comparisons were adjusted by Dunnett's T3 or Dunn's method to control for Type I errors. A two‐sided *p* < 0.05 indicated statistical significance: *p < 0.05, **p < 0.01, and ***p < 0.001.

## 3 Results

### 3.1 High FCGBP Expression Is Positively Associated With Increased Glioma Grade and Poor Patient Prognosis

**FIGURE 1:**
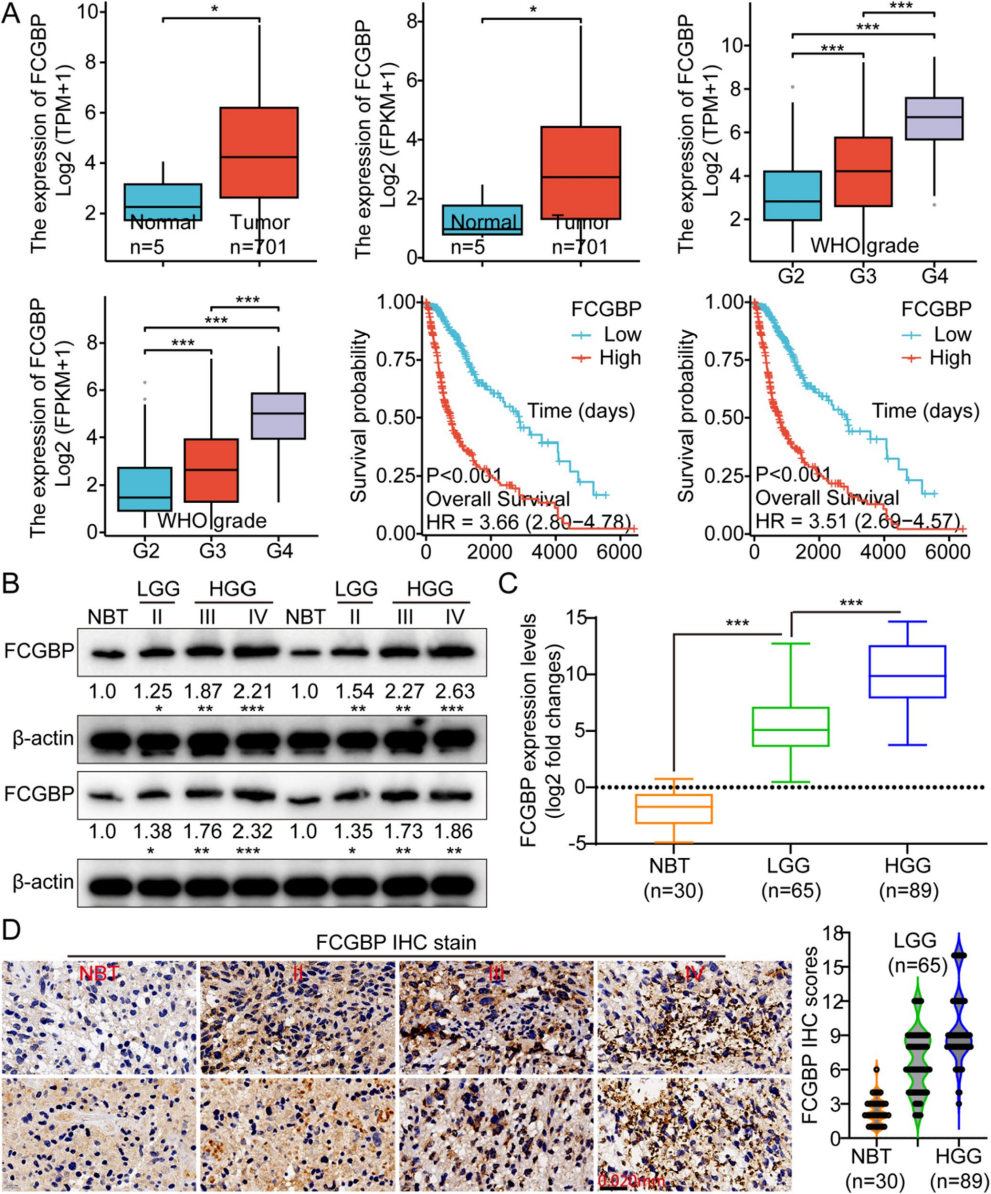
FCGBP is highly expressed in glioma and is negatively correlated with prognosis. (A) Analysis of the TCGA database revealed that FCGBP expression increased with tumor grade, and higher expression levels were associated with poorer prognosis. (B) WB analysis was performed to evaluate FCGBP expression in normal brain tissue and gliomas of different grades, including low‐grade glioma (LGG, grade II) and high‐grade glioma (HGG, grades III and IV). (C) FCGBP mRNA levels in normal brain tissue and various glioma grades were quantified using qRT‐PCR. (D) IHC staining and scoring were used to assess FCGBP expression in normal brain tissue and different glioma grades. Data were mean ± SD. Statistical significance was calculated by 2‐tailed unpaired Student's *t* tests and 1‐way ANOVA for A; 1‐way ANOVA for C and D; Survival analysis of A was performed by the log‐rank test. *p < 0.05, **p < 0.01, ***p < 0.001.

This study utilized the TCGA GM gene expression database, which contains genomic data from five normal brain tissues (NBTs) and 701 GM samples. Compared to NBTs, FCGBP was significantly upregulated in GM tissues and showed a positive relation with tumor grade while being inversely associated with patient prognosis (Figure 1A). Additionally, protein expression was analyzed using WB in GM tissue samples, including NBTs and GMs classified as II, III, and IV grades, confirming an elevated expression of FCGBP in GM tissues compared to NBTs. Furthermore, FCGBP overexpression was positively correlated with tumor grade at both the RNA and protein levels (Figure 1B,C). The results of IHC further demonstrated significant variations in the SI of FCGBP across diverse grades of GM. Subsequent quantitative analyses confirmed the recognized overexpression of the FCGBP protein (Figure 1D). Collectively, FCGBP may be a potential prognostic biomarker for GM.

### 3.2 FCGBP Overexpression Promotes Glioma Cell Proliferation, Migration, Invasion, and Accelerates Tumor Growth

**FIGURE 2:**
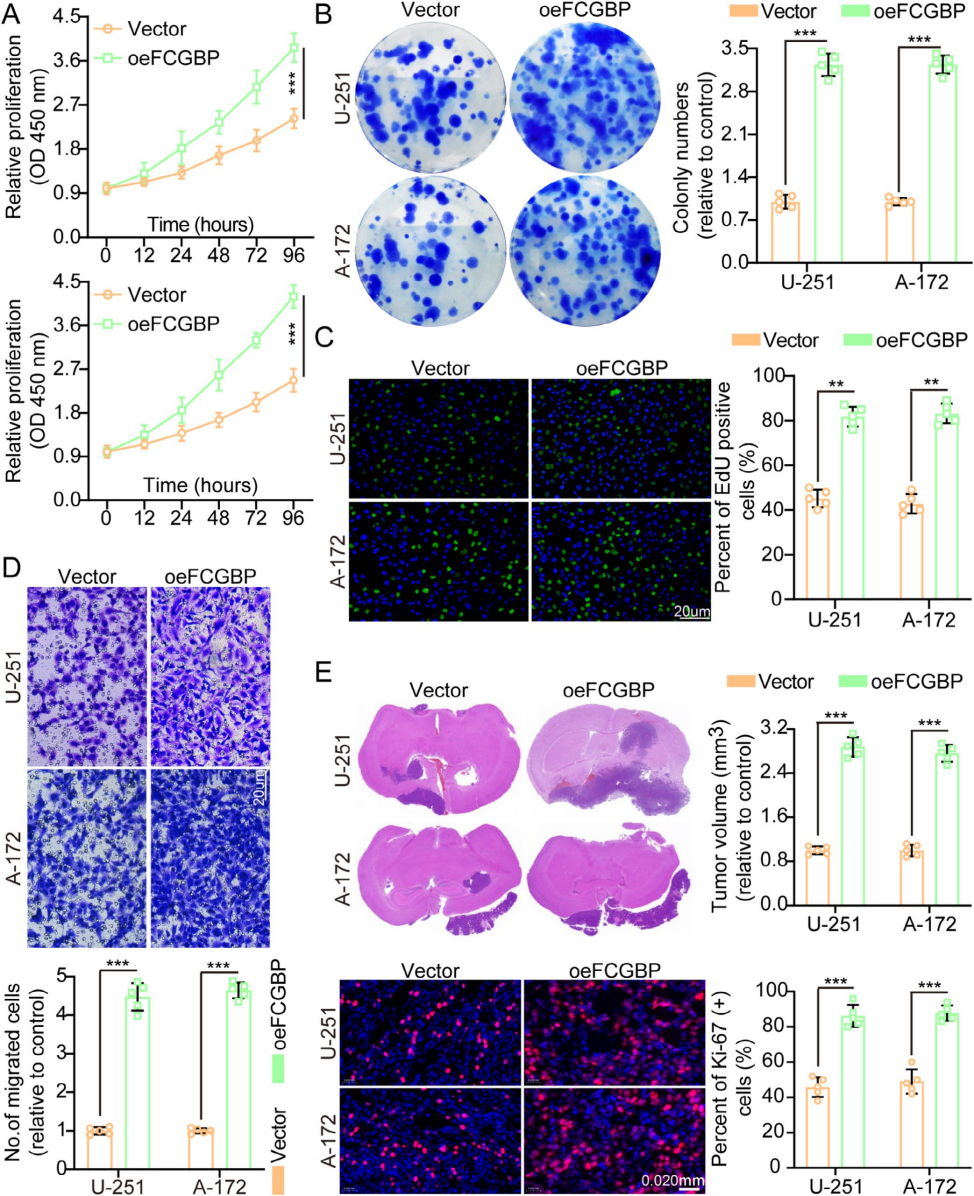
FCGBP overexpression enhances glioma cell aggressiveness. (A) Cell growth curves of Vector and oeFCGBP groups were assessed using the CCK‐8 assay (*n* = 5). (B) FCGBP overexpression promoted colony formation, as demonstrated by colony formation assays and corresponding histogram analysis (*n* = 5). (C) EdU incorporation assays indicated that FCGBP overexpression increased the number of EdU‐positive cells, suggesting enhanced cell proliferation (*n* = 5). (D) FCGBP upregulation facilitated cell migration, as shown by Transwell assays (*n* = 5; scale bars: 50 μm). (E) Tumor size and Ki‐67 staining were compared between Vector and oeFCGBP groups (*n* = 5). Data were mean ± SD. Statistical significance was calculated by 2‐way ANOVA for A; 2‐tailed unpaired Student's *t* tests for B–E. **p < 0.01, ***p < 0.001.

Initially, FCGBP expression levels were quantified via protein immunoblotting (WB) in normal human astrocytes (NHA) and eight GM cell lines, including U‐251, T98G, LN‐229, A‐172, LN‐18, H4, U118MG, and U‐87MG. The results revealed that GM cell lines exhibited significantly higher FCGBP protein levels compared to HA and NHA (Figure S1A). Based on these findings, U‐251 and A‐172 cells, which displayed moderate FCGBP expression, were selected for further investigation. The WB analysis confirmed the successful overexpression and knockdown of FCGBP in these two cell lines (Figure S1B,C). The CCK‐8, colony formation, TUNEL, and EdU results showcased that FCGBP overexpression significantly promoted GM cell growth (Figure 2A–C). Transwell assay findings indicated that FCGBP upregulation significantly enhanced the invasive and migratory capacities of GM cells (Figures 2D and S1D), suggesting its involvement in promoting malignant tumor progression.

In addition, in vivo experiments demonstrated that oeFCGBP facilitated tumor growth, as evidenced by Ki‐67 IF staining, which showed a higher proliferation index in the oeFCGBP group compared to the control (Figure 2E). Consistently, TUNEL assays revealed that FCGBP upregulation suppressed apoptosis (Figure S1F). Collectively, these results indicate that FCGBP overexpression not only inhibits cellular apoptosis but also accelerates tumor progression both in vitro and in vivo.

### 3.3 FCGBP Knockdown Suppresses Glioma Cell Malignancy and Enhances Tumor‐Suppressive Effects in Vivo

**FIGURE 3:**
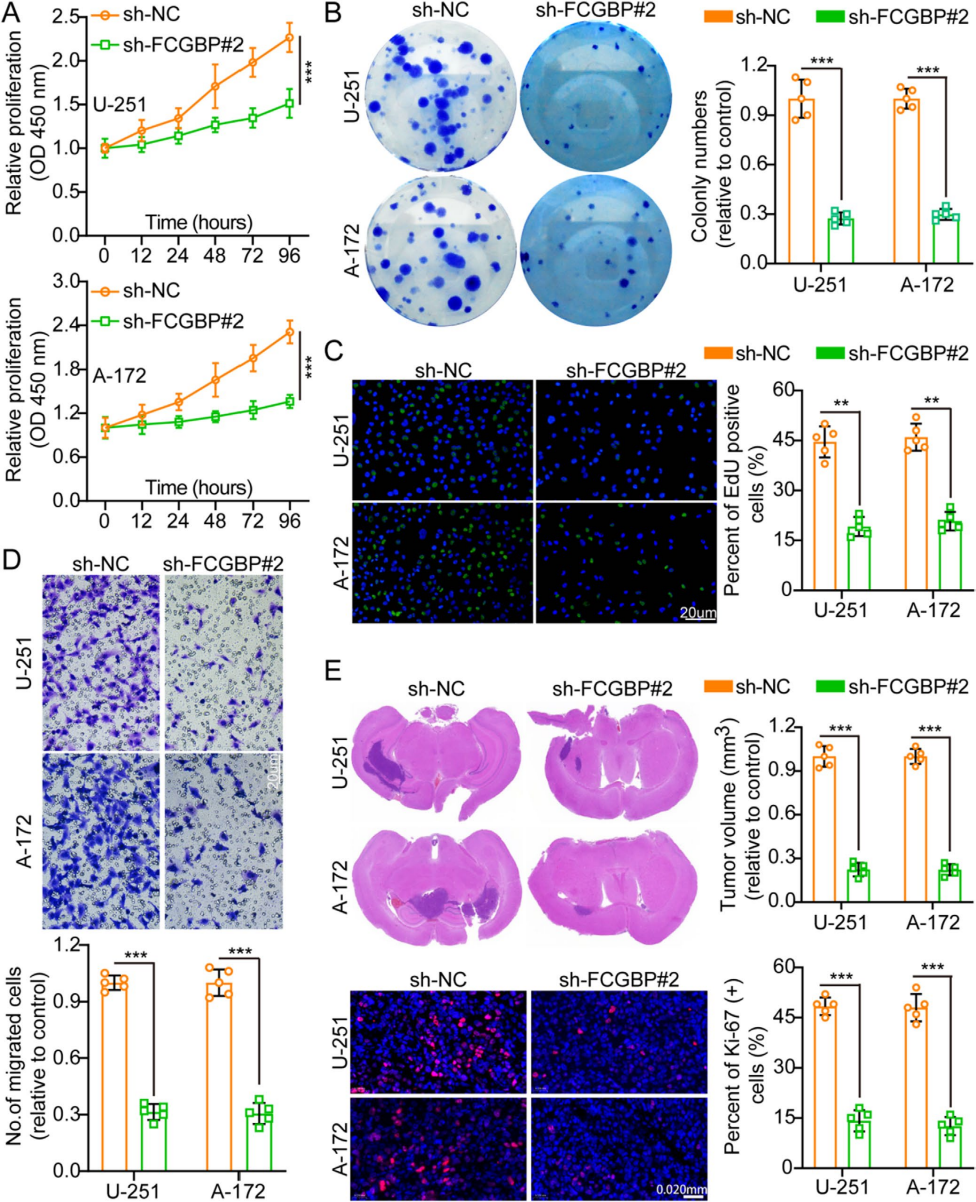
FCGBP knockdown suppresses glioma cell aggressiveness. (A) Cell growth curves of sh‐NC and sh‐FCGBP#2 groups were assessed using the CCK‐8 assay (*n* = 5). (B) Colony formation was significantly reduced after FCGBP knockdown, as shown by colony formation assays and corresponding histogram analysis (*n* = 5). (C) EdU incorporation assays revealed a decrease in EdU‐positive cells following FCGBP knockdown, indicating reduced cell proliferation (*n* = 5). (D) FCGBP knockdown impaired cell migration, as demonstrated by Transwell assays (*n* = 5; scale bars: 50 μm). (E) Tumor size and Ki‐67 staining were compared between sh‐NC and sh‐FCGBP#2 groups (*n* = 5). Data were mean ± SD. Statistical significance was calculated by 2‐way ANOVA for A; 2‐tailed unpaired Student's *t* tests for B–E. **p < 0.01, ***p < 0.001.

Herein, WB analysis confirmed the successful knockdown of FCGBP in U‐251 and A‐172 cells (Figure S1E). Among the tested shRNAs, sh‐FCGBP#2 exhibited the highest silencing efficiency and was used in subsequent experiments. Functional assays (CCK‐8, colony formation, EdU, and Transwell migration/invasion) demonstrated that FCGBP knockdown significantly attenuated GM cell aggressiveness (Figures 3A–C and S1E), highlighting its crucial role in tumor progression.

To further evaluate the impact of FCGBP suppression in vivo, stable FCGBP‐knockdown cell lines were implanted into nude mice and were sacrificed after 3 weeks, thereby analyzing brain tumors. IF staining revealed a significant reduction in Ki‐67 levels in the FCGBP‐knockdown group relative to the control (Figure 3E), indicating decreased proliferative activity. Additionally, TUNEL assays demonstrated that while FCGBP overexpression inhibits apoptosis, its knockdown significantly enhances apoptotic cell death (Figure S1F). These findings suggest that FCGBP suppression effectively impairs GM cell proliferation and promotes apoptosis in vivo.

### 3.4 FCGBP Activates the JAK2/STAT3/c‐Myc Signaling Axis to Promote Glioma Progression

Using the Cignal Finder Cancer 10‐Pathway Reporter Array, we screened for potential signaling pathways affected by FCGBP. Among the pathways analyzed, the JAK/STAT signaling axis was uniquely and significantly downregulated following FCGBP knockdown in U‐251 and A‐172 cells, whereas other pathways remained largely unchanged (Figure S2A). To further elucidate the downstream mechanisms mediated by FCGBP in glioma, we performed Gene Set Enrichment Analysis (GSEA) using KEGG and HALL‐MARK gene sets. The results revealed significant enrichment of inflammatory pathways—particularly the JAK–STAT signaling pathway—in samples with high FCGBP expression (Figure S2B,C). To validate these findings, we examined multiple inflammatory cytokines and observed a marked reduction in IL‐6 levels following FCGBP knockdown (Figure S2D). Consistently, qPCR and ELISA analyses further confirmed that suppression of FCGBP significantly decreased IL‐6 mRNA expression and protein secretion in U‐251 and A‐172 cell lines (Figure S2E).

**FIGURE 4:**
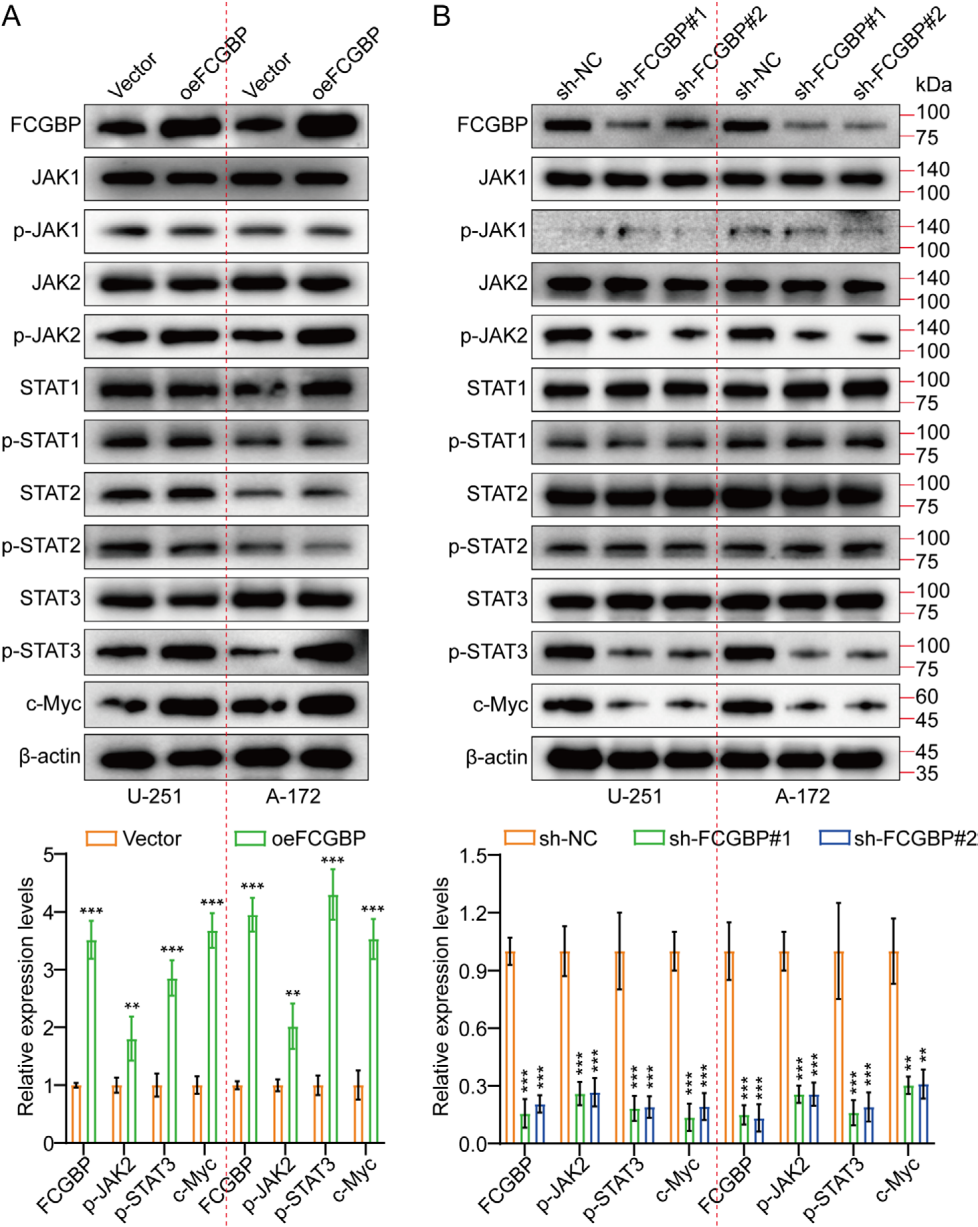
FCGBP Activates JAK2/STAT3 Signaling. (A, B) WB analysis was performed to evaluate the expression of FCGBP, a key component of the JAK2/STAT3 signaling pathway, and c‐Myc in Vector, oeFCGBP, sh‐NC, sh‐FCGBP#1, and sh‐FCGBP#2 groups. The results were further quantified and presented as a representative bar chart. Data were mean ± SD. Statistical significance was calculated by 2‐tailed unpaired Student's *t* tests for A; 1‐way ANOVA for B. **p < 0.01, ***p < 0.001.

To further verify the role of FCGBP in enhancing tumor growth via JAK/STAT pathway activation, we performed FCGBP knockdown and overexpression experiments in U‐251 and A‐172 cells, respectively. Herein, WB was conducted to assess JAK1/2 and STAT1/2/3, along with their phosphorylated forms (p), as well as c‐Myc expression. The results demonstrated that FCGBP suppression led to a significant reduction in p‐JAK2, p‐STAT3, and c‐Myc levels, whereas their expression was markedly upregulated upon FCGBP overexpression (Figure 4A,B). Additionally, analysis of TCGA data revealed a positive correlation between FCGBP and key signaling molecules, including JAK2, STAT3, and c‐Myc (Figure S3A). Altogether, FCGBP plays a pivotal role in activating the JAK2/STAT3/c‐Myc signaling axis, thereby contributing to tumor progression.

### 3.5 Pharmacologic Inhibition of JAK2/STAT3 Counteracts FCGBP‐Driven Glioma Progression

**FIGURE 5:**
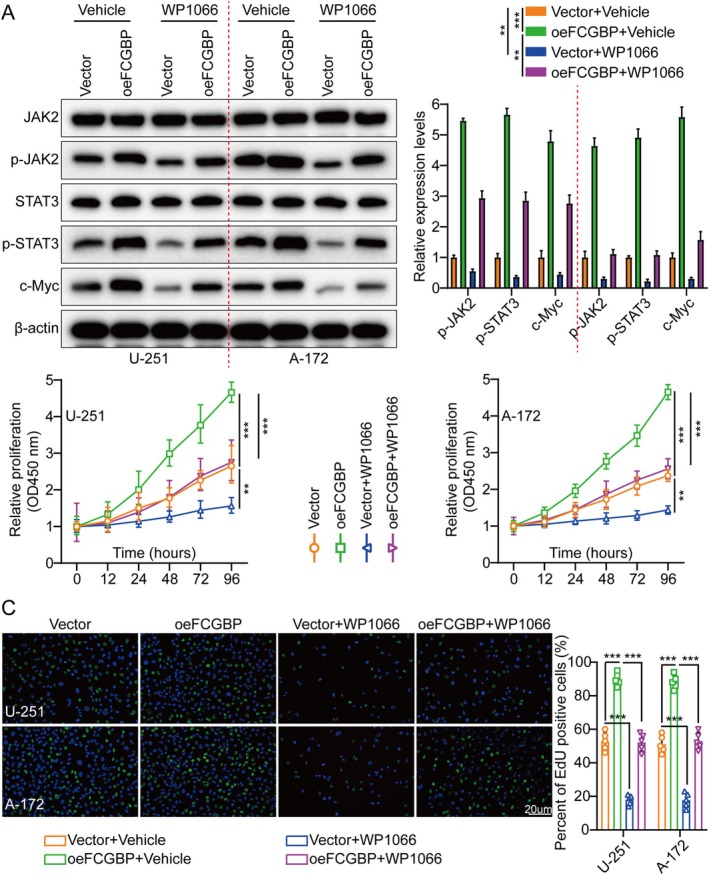
FCGBP promotes glioma growth by activating the JAK2/STAT3 signaling pathway. (A) WB analysis was performed to evaluate the expression levels of JAK2, p‐JAK2, STAT3, p‐STAT3, and c‐Myc in Vector, oeFCGBP, Vector + WP1066, and oeFCGBP + WP1066 groups. (B) Cell growth curves of different treatment groups were measured using the CCK‐8 assay. (C) EdU incorporation assay and corresponding histogram quantification were used to evaluate cell proliferation across different treatment conditions. Data were mean ± SD. Statistical significance was calculated by 2‐way ANOVA for A–C. **p < 0.01, ***p < 0.001.

To further elucidate the role of FCGBP in promoting cell proliferation by activating the JAK2/STAT3 signaling pathway, both U‐251 and A‐172 cells were treated with the JAK2/STAT3 inhibitor WP1066 (6 μM, 48 h). Additionally, the impact of FCGBP overexpression on cell proliferation was investigated. In line with prior findings, FCGBP overexpression significantly increased the levels of p‐JAK2, p‐STAT3, and c‐Myc, while total JAK2 and STAT3 expression remained unchanged. However, this effect was significantly attenuated when U‐251 and A‐172 cells were treated with WP1066 (Figure 5A). Moreover, functional assays and in vivo tumor models demonstrated that WP1066 effectively counteracted the pro‐tumorigenic effects of FCGBP, suppressing the aggressiveness of U‐251 and A‐172 cells (Figures 5B,C, S3B, and S4A,B). In alignment with in vitro findings, in vivo studies revealed that WP1066 treatment significantly inhibited tumor growth in GM‐bearing mice that had undergone cerebral orthotopic transplantation compared to the control groups. Notably, IF staining for Ki‐67 in xenograft tumors showed a substantial reduction in Ki‐67 expression in WP1066‐treated mice, suggesting that the inhibitor effectively mitigates FCGBP‐induced cell proliferation (Figure S4C). In addition, the TUNEL test results also verified the above conclusions (Figure S5A).

Collectively, FCGBP promotes GM progression by inducing JAK2/STAT3 signaling. Targeting this pathway with WP1066 may be a possible GM therapeutic strategy.

### 3.6 FCGBP Drives Glioma Progression Through JAK2/STAT3‐Dependent Upregulation of c‐Myc

**FIGURE 6:**
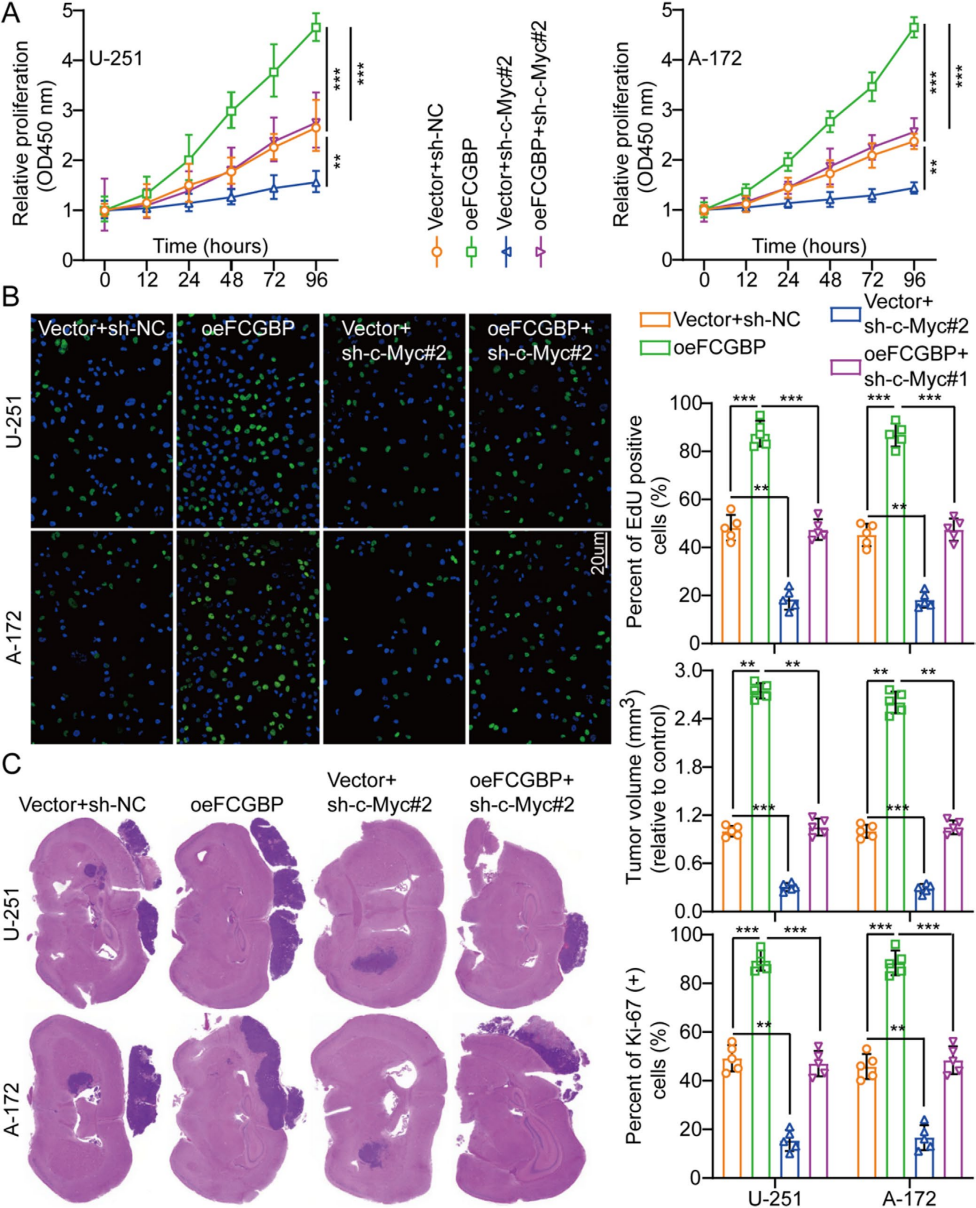
FCGBP promotes glioma progression by activating the JAK2‐STAT3‐c‐Myc signaling pathway. (A) Cell growth curves in the different treatment groups were assessed using the CCK‐8 assay (*n* = 5). (B) The EdU assay and corresponding histogram analysis were performed to evaluate cell proliferation across various treatment groups (*n* = 5). (C) Tumor weight histograms and frozen sections of mouse brain tissue were analyzed for each treatment group (*n* = 5). Data were mean ± SD. Statistical significance was calculated by 2‐way ANOVA for A–C. **p < 0.01, ***p < 0.001.

Normal cellular function depends on c‐Myc proteins, which regulate gene expression associated with the cell cycle, proliferation, and apoptosis. The c‐Myc mutations or overexpression disrupt cell growth regulation and are crucial in the development of cancer. The MYC gene is frequently hyperactive in various tumors [17, 18]. In U‐251 and A‐172 cell lines, shRNAs targeting c‐Myc were deployed to explore their implication in FCGBP‐modulated GM cell proliferation. Among the candidates, sh‐c‐Myc#2 displayed the highest knockdown efficiency and was selected for subsequent analyses (Figure S5B). Previous findings also revealed that c‐Myc expression decreased upon FCGBP knockdown and increased with FCGBP overexpression (Figure 4A,B). To assess whether FCGBP regulates c‐Myc expression via JAK2/STAT3 signaling, FCGBP was overexpressed, and cells were treated with the JAK2/STAT3 inhibitor WP1066. Protein analysis by WB demonstrated that FCGBP overexpression led to an increase in c‐Myc levels, while WP1066 treatment attenuated this effect (Figure 5A). Additionally, c‐Myc suppression reduced FCGBP overexpression‐induced proliferative, migratory, and invasive effects in U‐251 and A‐172 cells (Figures 6A,B, S5C, and S6A).

In an intracranial orthotopic GM mouse model, these in vivo findings aligned with in vitro observations, showing that c‐Myc silencing significantly attenuated the FCGBP‐induced enhancement of tumor growth compared to control (Figure 6C). Moreover, c‐Myc knockdown effectively mitigated FCGBP‐driven cell proliferation, as evidenced by the reduced Ki‐67 expression in xenograft tumors within the c‐Myc silencing groups (Figure S6B). Furthermore, TUNEL assay results indicated that FCGBP upregulation inhibited cell apoptosis, whereas c‐Myc knockdown counteracted this effect (Figure S6C). Collectively, these findings suggest that FCGBP promotes GM progression by activating the JAK2/STAT3/c‐Myc signaling pathway.

### 3.7 HIF‐1α Directly Binds to and Transactivates the FCGBP Promoter in Glioma

**FIGURE 7:**
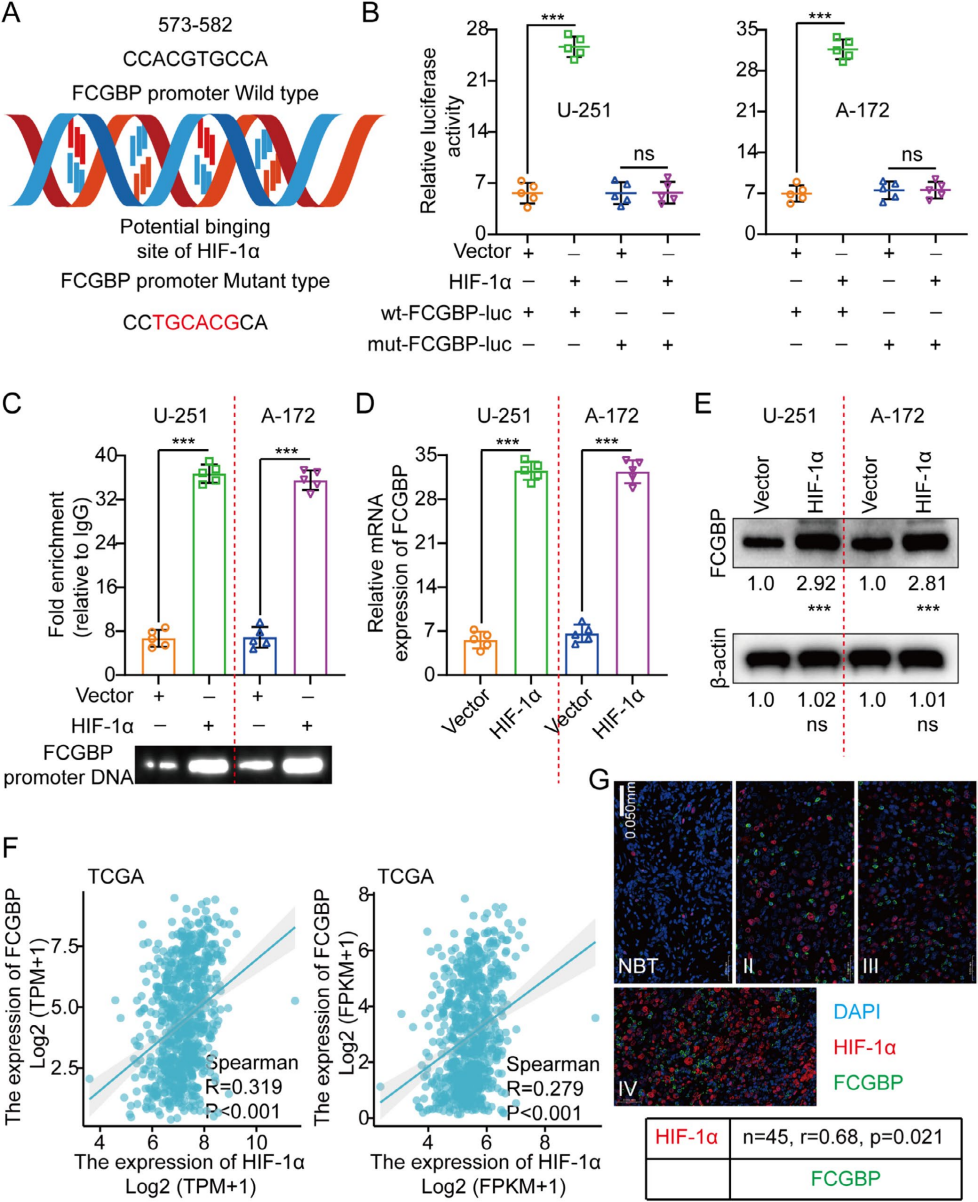
FCGBP is a direct target of HIF‐1α. (A) Based on the predicted binding site of HIF‐1α on the FCGBP promoter, wild‐type and mutant luciferase reporter vectors were constructed. (B) Luciferase activity was measured by transfecting U‐251 and A‐172 cells with either wild‐type or mutant luciferase vectors, followed by co‐transfection with empty or HIF‐1α expression plasmids. (C) ChIP assays were performed to investigate the role of HIF‐1α in gene regulation, using IgG as an internal control for specificity. PCR amplification was conducted using primers targeting the FCGBP promoter to detect HIF‐1α‐bound DNA. (D, E) FCGBP expression was analyzed using qRT‐PCR and WB following exogenous HIF‐1α overexpression. (F) TCGA database analysis revealed a positive correlation between HIF‐1α and FCGBP expression. (G) Immunofluorescence staining and immunohistochemical scoring in clinical samples confirmed a positive correlation between HIF‐1α and FCGBP expression. Ns, no significant difference. Data were mean ± SD. Statistical significance was calculated by 2‐way ANOVA for B; 2‐tailed unpaired Student's *t* tests for C and D; Spearman's rank correlation test for F. **p < 0.01, ***p < 0.001.

In GMs, hypoxia plays a vital role in shaping the tumor microenvironment, frequently driving invasion, metastasis, and malignancy [19, 20]. Transcription factors govern gene expression by binding to specific DNA sequences. According to the JASPAR database [21], HIF‐1α is predicted to bind to the FCGBP gene promoter region. To investigate whether HIF‐1α regulates FCGBP transcription, we introduced luciferase reporter vectors harboring either the wild‐type (WT) or mutant (mut) FCGBP promoter into U‐251 and A‐172 cells (Figure 7A). The luciferase results showcased that HIF‐1α overexpression significantly enhanced WT FCGBP promoter activity, as indicated by increased luciferase activity. Nonetheless, the mut FCGBP promoter exhibited no response to HIF‐1α overexpression (Figure 7B). Chromatin immunoprecipitation (ChIP) assays further confirmed direct binding of HIF‐1α to the FCGBP promoter (Figure 7C), while its upregulation resulted in increased FCGBP expression (Figure 7D,E). Additionally, TCGA analysis and IF staining further validated a positive correlation between HIF‐1α and FCGBP expression in GM tissues (Figure 7F,G). In addition, pan‐cancer analysis using the TCGA database also showed that FCGBP is specifically highly expressed in most tumors (Figure S7). To further demonstrate that HIF‐1α is the only transcription factor regulating FCGBP expression in glioma, we conducted additional experiments under hypoxic conditions (e.g., CoCl_2_ treatment) to support the above point. The results showed that, compared to normoxic treatment, both hypoxia and CoCl_2_ treatment were able to upregulate the expression level of FCGBP (Figure S8A).

While our current study does not include patient‐level treatment‐response data to JAK2/STAT3 inhibitors (which are not part of standard‐of‐care for glioma in our clinical setting, and are not systematically annotated in the public cohorts analyzed), we have strengthened the clinical relevance by incorporating pathway‐activity‐based evidence in large independent datasets. Specifically, using TCGA and CGGA transcriptomic cohorts, we quantified STAT3 pathway activity by calculating a STAT3‐associated gene signature score (e.g., GSVA/ssGSEA based on published STAT3 target gene sets). We found that FCGBP expression is positively correlated with STAT3 signature scores across both cohorts (Figure S8B,C). Consistently, tumors with high FCGBP expression showed significantly higher STAT3 pathway scores compared with FCGBP‐low tumors. In addition, GSEA demonstrated that JAK/STAT3 signaling and related oncogenic programs (including c‐Myc‐associated gene sets) are enriched in FCGBP‐high gliomas.

## 4 Discussion

Typically, GMs are tumors originating from glial cells, which are indispensable in supporting and protecting neurons within the CNS. These tumors are classified based on their cellular origin and malignancy grade [3, 22]. The most common subtypes include astrocytomas, ependymomas, and oligodendrogliomas. Depending on their malignancy, GMs are further categorized into low‐grade and high‐grade tumors. Among them, high‐grade GMs, particularly GBM, represent the most aggressive and prevalent malignant brain tumors, characterized by extensive invasiveness and poor prognosis [23, 24]. The pathogenesis of GMs is highly complex, involving multiple contributing factors, including genetic mutations, alterations in the tumor microenvironment, and metabolic reprogramming. Current treatment strategies primarily include surgical resection, chemotherapy, and radiotherapy. However, due to the highly invasive nature of GMs and their high recurrence rate following treatment, patient survival remains significantly limited [25]. Therefore, the exploration of novel therapeutic approaches, including immunotherapy, targeted therapy, and gene therapy, is crucial for improving treatment outcomes and enhancing patient prognosis.

FCGBP is a mucin‐associated protein that is highly expressed in various epithelial tissues, particularly in mucus‐secreting cells of the gastrointestinal, respiratory, and urinary tracts [26]. FCGBP primarily forms high‐molecular‐weight complexes through disulfide bonds and, together with components such as MUC2 (mucin 2), contributes to the epithelial barrier, helping to maintain the mucus barrier function in the intestine and respiratory tract [27]. Recently, the role of FCGBP in maintaining the mucus barrier, promoting intestinal homeostasis, and influencing tumor occurrence and development has gradually garnered attention. For example, FCGBP forms a complex with mucin MUC2 and members of the trefoil factor family, inhibiting bacterial adhesion to the mucosal surface, affecting pathogen motility, and facilitating their clearance [27]. This function is crucial for maintaining the integrity of the intestinal mucus layer. Multiple studies have manifested that FCGBP is abnormally expressed in various cancers. For example, in GMs, high FCGBP expression is associated with a poor prognosis, and its expression level is closely linked to various immune cell infiltrations [8, 11]. Additionally, in CRC, decreased FCGBP expression is associated with tumor progression and reduced patient survival, suggesting its potential role as a novel regulatory factor in CRC [28]. Interestingly, Pan‐Cancer Database analysis showed that FCGBP was significantly overexpressed in most tumors (Figure S7). Due to the expression changes of FCGBP in various diseases, it is considered to have potential as a diagnostic and prognostic biomarker. For example, in CRC, FCGBP expression in circulating tumor cells has been studied as a potential biomarker. Overall, FCGBP is significant in mucosal immunity and tumor biology, and its potential as a biomarker warrants further investigation.

The JAK2‐STAT3‐MYC signaling is critical in cell proliferation, differentiation, apoptosis, and immune regulation. Recently, this pathway has been extensively studied, particularly in relation to tumorigenesis and cancer progression. JAK2‐STAT3 signaling is a component of the JAK–STAT signaling pathway, where cytokines or growth factors bind to cell membrane receptors, activating the JAK2 kinase and subsequently activating STAT3. Activated STAT3 undergoes nuclear translocation, where it regulates the transcription of specific genes, such as the anti‐apoptotic gene Bcl‐xL and the proliferation‐related gene Cyclin D1 [9]. In various cancers, the JAK2‐STAT3 pathway is abnormally activated. This persistent activation promotes tumor cell proliferation, inhibits apoptosis, and enhances invasion and metastasis. For example, sustained STAT3 activation in non‐small cell lung cancer (NSCLC) has been reported to be related to increased tumor growth and invasiveness [29].

Additionally, STAT3 activation can upregulate PD‐L1 expression, leading to immune evasion and further promoting tumor resistance to therapy [30, 31]. MYC is a key transcription factor governing cell growth and metabolism. Studies have demonstrated that JAK2‐STAT3 signaling can upregulate MYC expression through both direct and indirect mechanisms [32]. In hepatocellular carcinoma (HCC), excessive activation of STAT3 can promote MYC expression, thereby driving tumor development and progression [33]. Furthermore, STAT3 may interact with the protein kinase DNA‐activated catalytic polypeptide (PRKDC) to activate the MYC signaling pathway, thereby promoting PD‐L1 expression and inducing immune evasion [34]. Given the critical role of the JAK2‐STAT3‐MYC axis in cancer, therapeutic strategies targeting this pathway have attracted significant attention. JAK2 and STAT3 inhibitors have shown potential in suppressing tumor growth in various cancer models. For instance, the JAK2 inhibitor AG490 can block the JAK–STAT pathway, thereby hampering tumor cell proliferation and inducing apoptosis [35]. Additionally, therapeutic strategies targeting MYC are actively being explored to halt tumor progression by inhibiting MYC function. In summary, JAK2‐STAT3‐MYC signaling is crucial in the biology of tumors. Further research into its mechanisms will contribute to the development of novel therapeutic strategies and improve patient prognosis.

HIF‐1α is an important transcription factor that enables cellular adaptation to low oxygen conditions (hypoxia). In tumor microenvironments, hypoxia is common due to rapid cell proliferation outpacing blood supply, leading to increased HIF‐1α expression. This overexpression is associated with tumor progression and metastasis. HIF‐1α regulates genes involved in angiogenesis, metabolism, and survival, thereby facilitating tumor growth under hypoxic conditions [36, 37]. Notably, it upregulates vascular endothelial growth factor, enhancing new blood vessel formation to supply the tumor. HIF‐1α also alters cellular metabolism by increasing the expression of enzymes involved in glycolysis, enabling tumor cells to thrive despite low oxygen levels. Elevated HIF‐1α levels have been observed in gastric, colon, breast, pancreatic, kidney, prostate, ovarian, brain, and bladder cancers. High HIF‐1α expression often correlates with aggressive tumor behavior and poor prognosis. It serves as a predictive marker for resistance to treatments such as radiation and chemotherapy [38, 39]. For instance, in breast cancer, elevated HIF‐1α levels are linked to increased microvascular density and reduced patient survival rates [40].

Given its role in tumor biology, HIF‐1α is a potential therapeutic target. Researchers are investigating HIF inhibitors, such as phenethyl isothiocyanate and acriflavine, for their anti‐cancer effects. However, developing effective HIF‐1α‐targeted therapies remains challenging due to the complex nature of cancer and the need for specificity to avoid affecting normal cellular functions. In summary, HIF‐1α plays a pivotal role in tumor development and progression by enabling cancer cells to adapt to hypoxic environments. Ongoing research aims to harness this knowledge for improved diagnostic and therapeutic strategies in oncology.

Our findings indicate that FCGBP is distinctly overexpressed in GM and is strongly associated with individual prognosis. Mechanistically, HIF‐1α directly regulates FCGBP expression, leading to its upregulation and subsequently driving GM progression by activating JAK2/STAT3/c‐Myc signaling (Figure S9). These results highlight the potential of FCGBP as a promising target for gene therapy and underscore the need to clarify its precise molecular mechanisms and evaluate its therapeutic significance.

Of course, the findings of this study remain preliminary and somewhat speculative, as this is one of the first investigations to systematically explore FCGBP in the context of gliomas. We also emphasize the necessity of future research, including external validation and clinical studies, to further substantiate our results and strengthen the evidence supporting FCGBP as a potential therapeutic target.

## Author Contributions

Jin Zheng, Yu xin Rao, Hui Zheng, and Liang liang Shi conceptualized the study and authored the manuscript; Yu xin Rao and Jin Zheng collected clinic samples; Jin Zheng, Yu xin Rao, and Liang liang Shi conducted the experiment and data analysis.

## Funding

The Natural Science Foundation of Hubei, China (no. 2023AFB740).

## Ethics Statement

The Ethical Board at Wuhan Union Hospital reviewed and authorized this investigation.

## Conflicts of Interest

The authors declare no conflicts of interest.

## Supporting information


**Data S1:** cam471617‐sup‐0001‐supinfo.docx.

## Data Availability

The data that support the findings of this study are available from the corresponding author upon reasonable request.
