# Supplementary material for: FCGBP Promotes Glioma Growth by Regulating JAK2/STAT3/c‐Myc Pathway
Source: Cancer Med. 2026 Feb 15;15(2):e71617. doi: 10.1002/cam4.71617 (PMC12906970; doi:10.1002/cam4.71617)
Supplement: Supplementary file 1 — Data S1: cam471617‐sup‐0001‐supinfo.docx. [file CAM4-15-e71617-s001.docx]

**FCGBP Promotes Glioma Growth by Regulating JAK2/STAT3/c-Myc Pathway**

Jin Zheng^1#^, Yu xin Rao^1#^, Hui Zheng^2*^, Liang liang Shi^3*^

**Materials and Methods**

**Cignal finder cancer 10-pathway reporter array**

All procedures were carried out in accordance with the manufacturer's instructions. Cells were resuspended and seeded into 96-well plates along with luciferase reporters targeting key cancer-related pathways. After incubation, luciferase activity was measured to assess pathway activation.

**Real-time quantitative RT-PCR (qRT-PCR)**

Total RNA was extracted from cellular and tissue samples using TRIzol and TRIzol LS reagents (Life Technologies). MicroRNAs (miRNAs) were then reverse transcribed with the Mir-X™ miRNA First-Strand Synthesis Kit (Clontech, Mountain View, CA, USA). Quantitative real-time polymerase chain reaction (qRT-PCR) was conducted using the SYBR Green PCR Master Mix (Takara, Shiga, Japan) and primers listed in Table S3. Messenger RNA (mRNA) expression levels were quantified with the 7500 Fast Real-Time PCR System (Applied Biosystems, Foster City, CA, USA), using glyceraldehyde 3-phosphate dehydrogenase (GAPDH) as the internal reference gene.

**Table S1: Related antibodies involved**

| Name | Description |
| --- | --- |
| Anti-FCGBP antibody | [NBP3-42011](https://www.bio-techne.com/p/antibodies/fcgbp-antibody_nbp3-42011) (Bio-Techne, Minneapolis, USA) |
| Anti-β-Actin antibody | #8457 (Cell Signaling Technology, Beverly, MA, USA) |
| Anti-JAK1 antibody | ab133666 (Abcam, Cambridge, USA) |
| Anti-p-JAK1 antibody | ab138005 (Abcam, Cambridge, USA) |
| Anti-JAK2 antibody | ab108596 (Abcam, Cambridge, USA) |
| Anti-p-JAK2 antibody | ab32101 (Abcam, Cambridge, USA) |
| Anti-STAT1 antibody | ab234400 (Abcam, Cambridge, USA) |
| Anti-p-STAT1 antibody | ab109461 (Abcam, Cambridge, USA) |
| Anti-STAT2 antibody | ab32367 (Abcam, Cambridge, USA) |
| Anti-p-STAT2 antibody | ab191601 (Abcam, Cambridge, USA) |
| Anti-STAT3 antibody | ab68153 (Abcam, Cambridge, USA) |
| Anti-p-STAT3 antibody | ab267373 (Abcam, Cambridge, USA) |
| Anti-c-Myc antibody | 10828-1-AP (Proteintech, Wuhan, China ) |
| Anti-HIF-1α antibody | 20960-1-AP (Proteintech, Wuhan, China ) |
| Anti-Ki-67 antibody | #9129 (Cell Signaling Technology, Beverly, MA, USA) |

**Table S2: The specific sh-RNAs sequences utilized in this study**

| **Gene** | **Primer** | **Sequence(5′-3′)** |
| --- | --- | --- |
| sh-FCGBP#1 | forward | CCCTTGAAAGATTGCATCTTT |
| sh-FCGBP#2 | forward | CCTGTAACTATGTGCTGGCAA |
| sh-c-Myc#1 | forward | TAATGATAACCAGAACTTGCT |
| sh-c-Myc#2 | forward | CACCACCAGCACACGGAACTA |

**Table S3: The Primer sequences in this study**

| **Gene** | **Primer** | **Sequence(5′-3′)** |
| --- | --- | --- |
| FCGBP | forward | GCCAAGGCTGAGATGATAGGC |
|  | reverse | CCTGCACAGAGATGGCATAGT |
| c-Myc | forward | GGCTCCTGGCAAAAGGTCA |
|  | reverse | CTGCGTAGTTGTGCTGATGT |
| GAPDH | forward | GAGTCAACGGATTTGGTCGT |
|  | reverse | TTGATTTTGGAGGGATCTCG |

**Figure Legends**

**
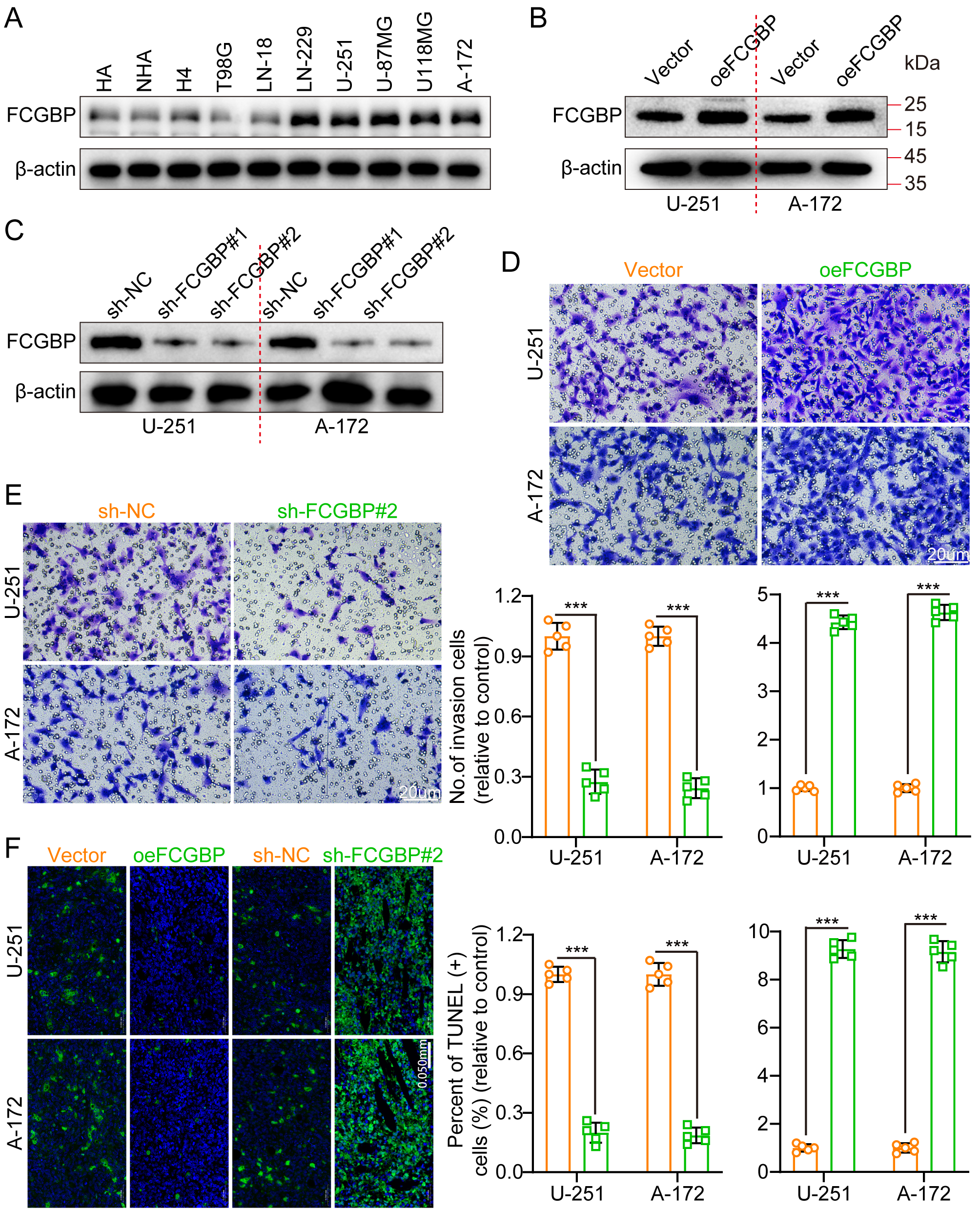
**

**Supplementary Figure 1: Functional experiments of FCGBP.**

**A.** Western blot analysis of FCGBP expression across various cell lines, including HA, NHA, U-251, T98G, LN-229, A-172, LN-18, H4, U118MG, and U-87MG. **B-C.** Validation of FCGBP overexpression and knockdown efficiency using Western blot. **D.** Transwell invasion assays demonstrate that FCGBP overexpression enhances cell invasion (n=5). Scale bars: 50 µm. **E.** Transwell invasion assays indicate that FCGBP knockdown reduces cell invasion (n=5). Scale bars: 50 µm. **F.** Representative images of TUNEL staining across different treatment groups (n= 5). Scale bars: 0.050 mm. Data were mean ± SD. Statistical significance was calculated by 2-tailed unpaired Student’s *t* tests for **E** and **F**. ****P* < 0.001.


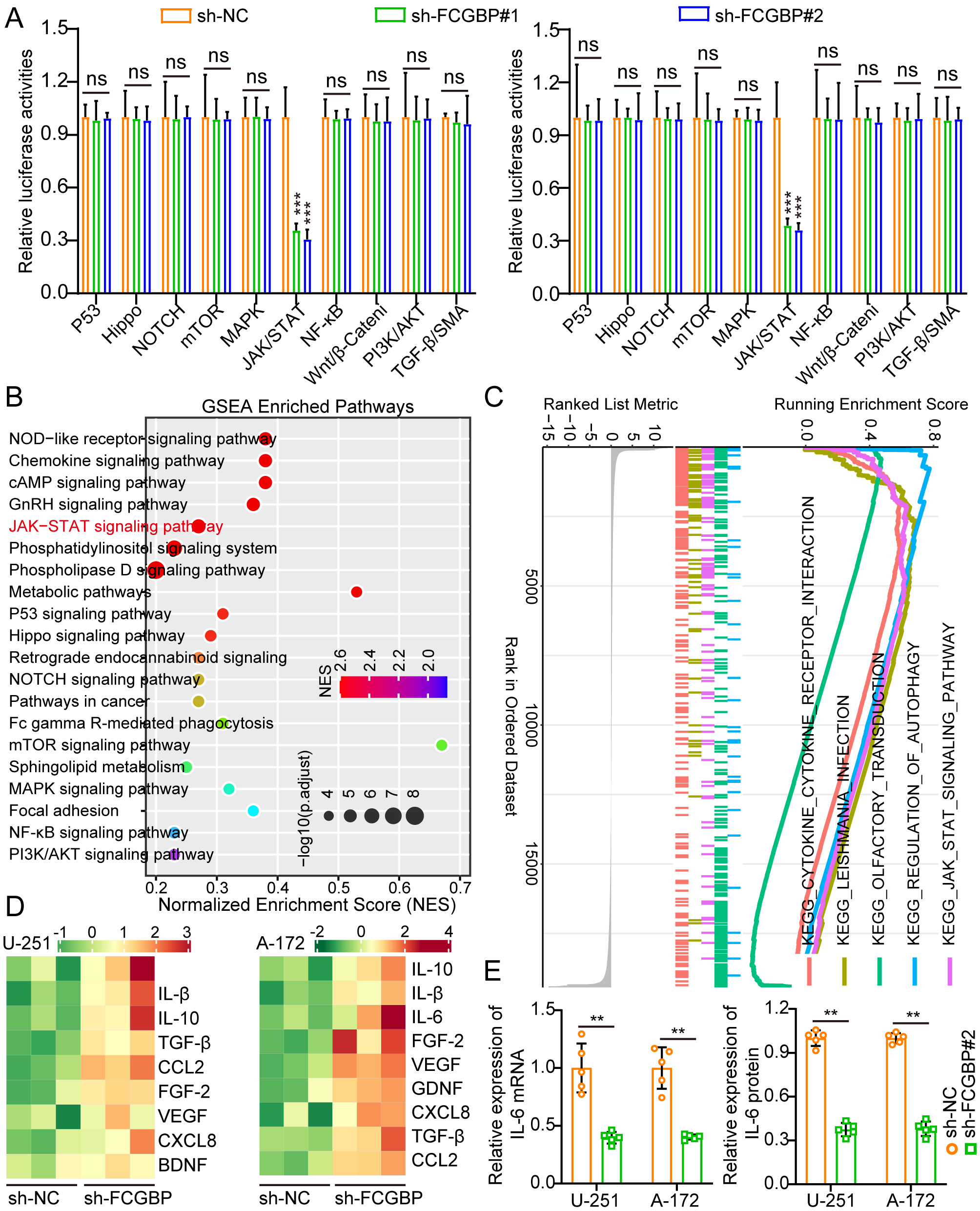


**Supplementary Figure 2: FCGBP initiates the JAK2/STAT3 signaling pathway.**

**A.** The Cignal Finder Cancer 10-Pathway Reporter Array demonstrated that FCGBP knockdown significantly suppressed the JAK/STAT signaling pathway in U-251 and A-172 cells, whereas other pathways remained unaffected. **B-C.** Gene Set Enrichment Analysis (GSEA) identified a strong association between FCGBP and the JAK/STAT signaling pathway. Data were mean ± SD. Statistical significance was calculated by 1-way ANOVA for **A**; 2-tailed unpaired Student’s *t* tests for **E**. ***P* < 0.01, ***P < 0.001. NS: Not significant.


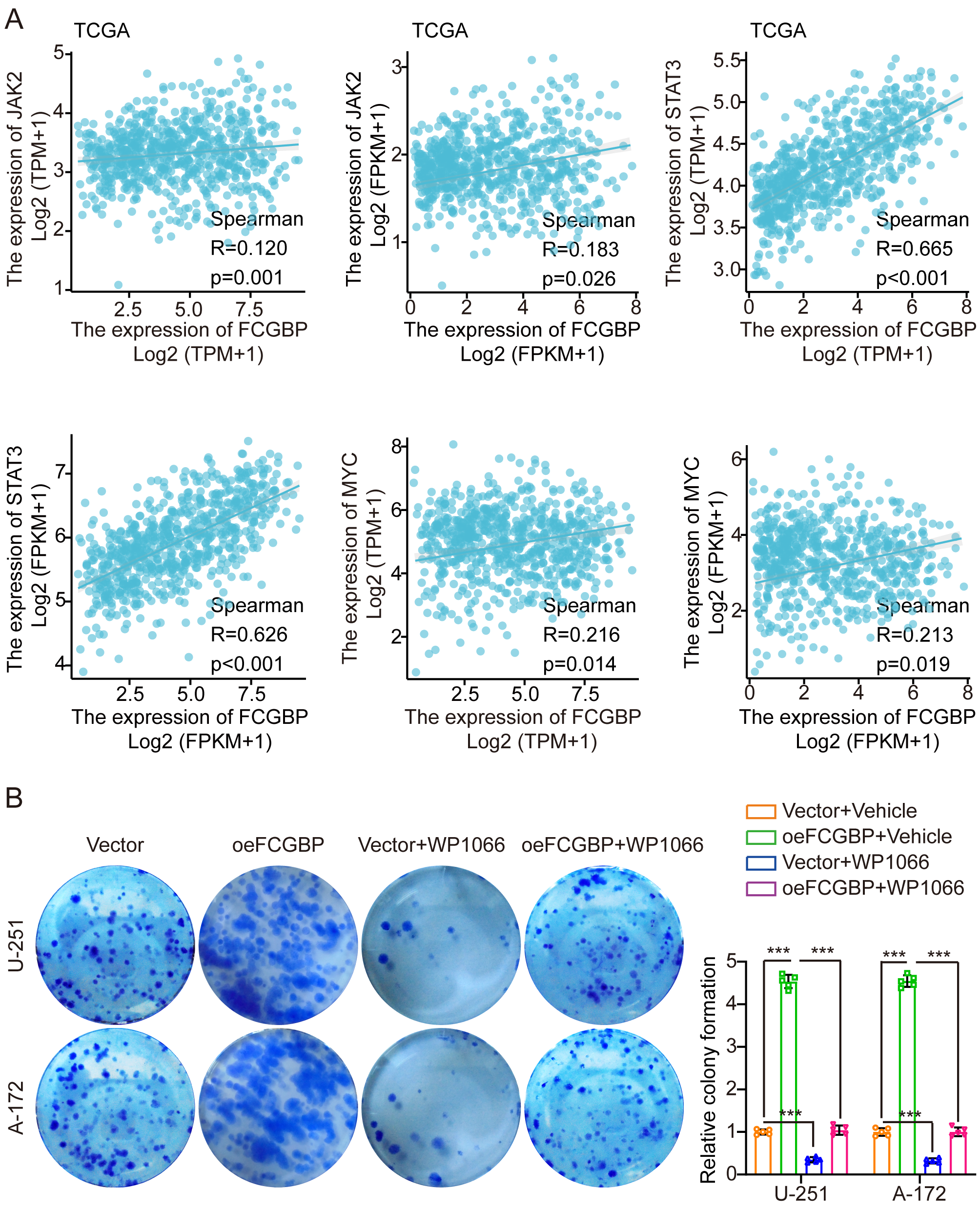


**Supplementary Figure 3: A positive correlation exists between FCGBP and molecules related to the JAK2/STAT3/c-Myc signaling pathway.**

**A.** TCGA database analysis revealed a positive correlation between FCGBP expression and key molecules involved in the JAK2/STAT3/c-Myc signaling pathway. **B.** Colony formation assays, along with histogram quantification, were performed to assess cell growth across different treatment groups. Data were mean ± SD. Statistical significance was calculated by Spearman’s rank correlation test for **A**; 2-way ANOVA for **B**. ****P* < 0.001.


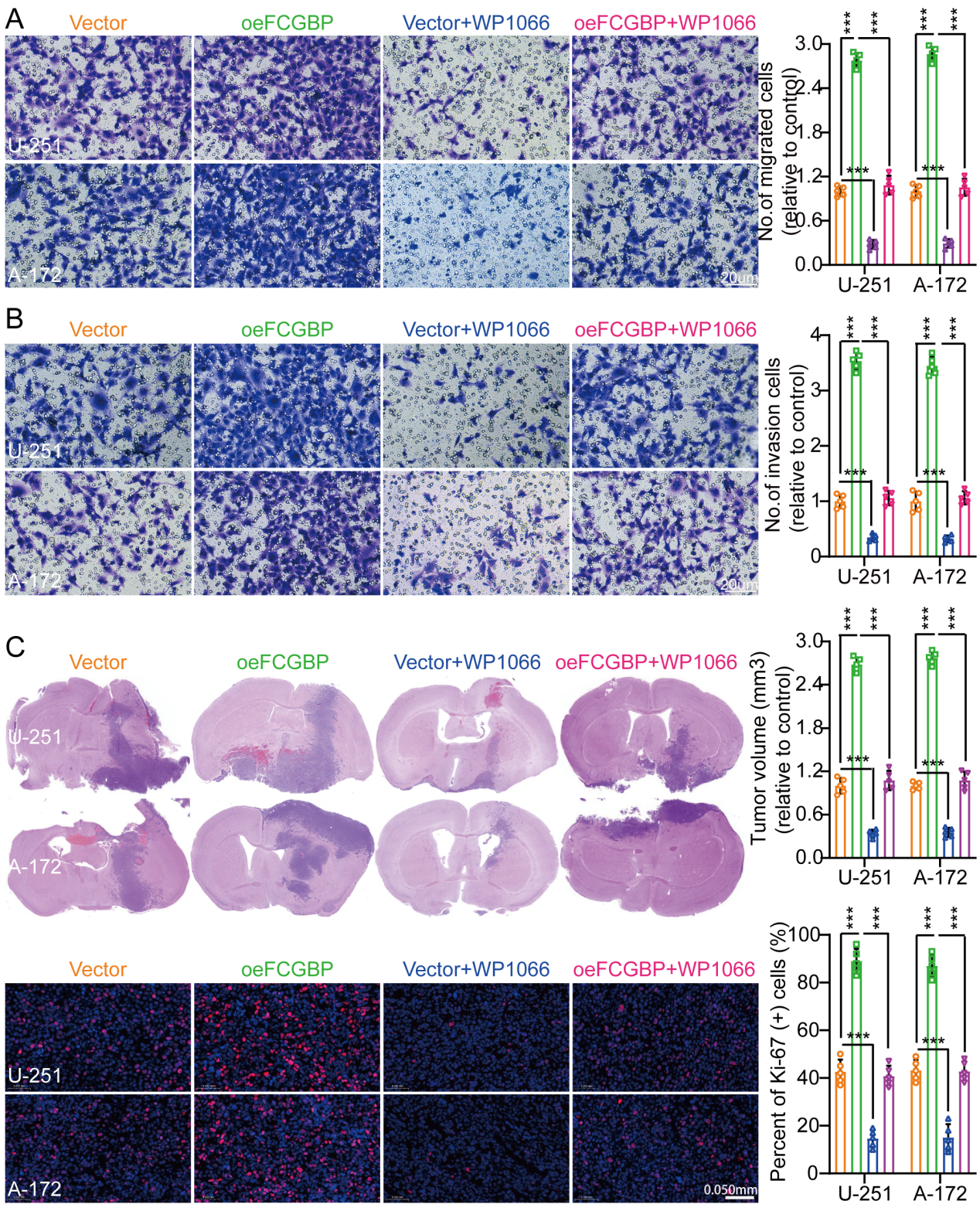


**Supplementary Figure 4: FCGBP enhances progression by triggering the JAK2/STAT3 signaling pathway.**

**A-B.** The malignant phenotype of cells across different treatment groups was evaluated using Transwell migration/invasion assays, with corresponding histogram quantification. **C.** Representative images of Ki-67 staining and frozen sections of mouse brain tissue for each treatment group. Data were mean ± SD. Statistical significance was calculated by 2-way ANOVA for **A-C**. ****P* < 0.001.


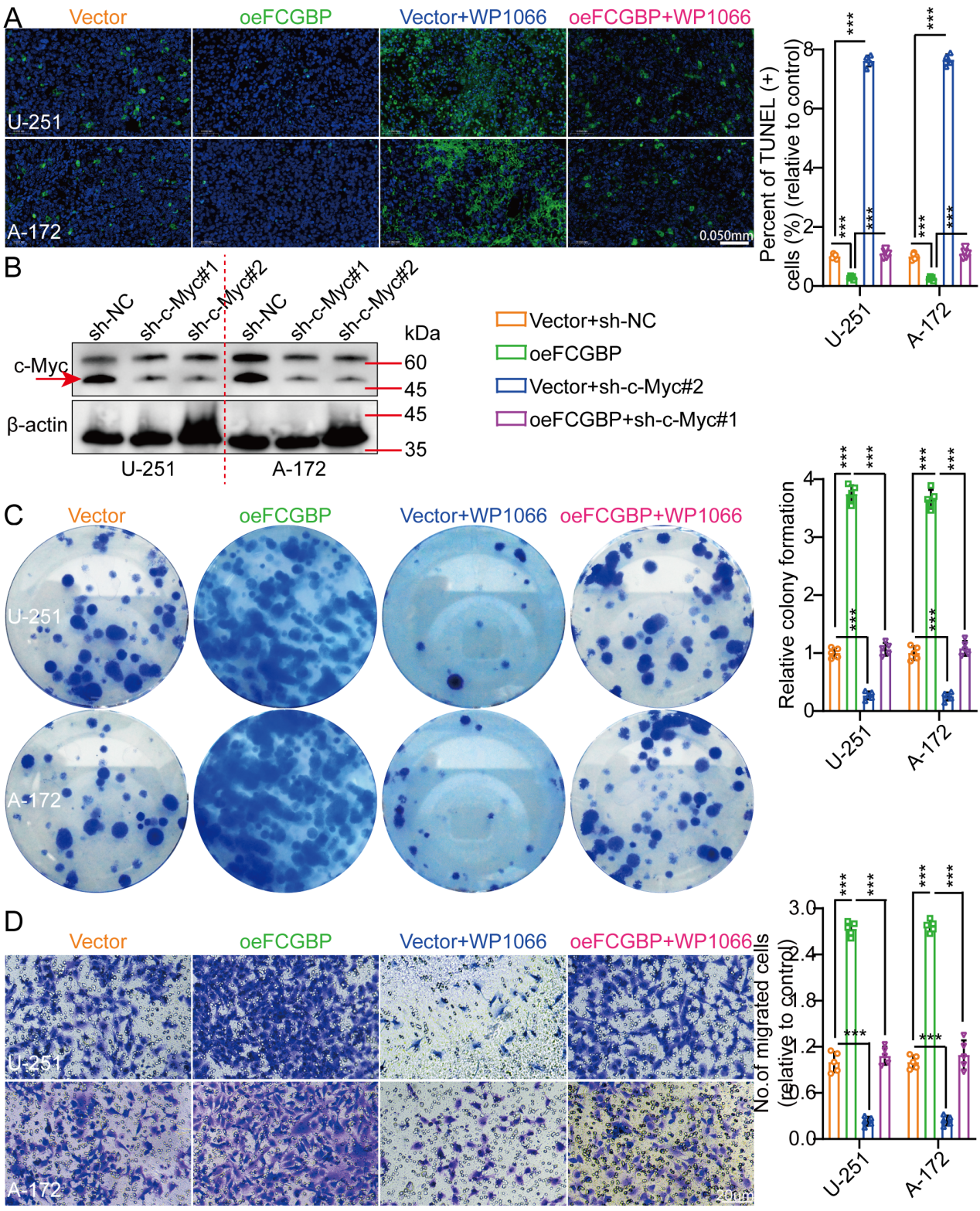


**Supplementary Figure 5: FCGBP enhances glioma development by activating the JAK2-STAT3-c-Myc signaling pathway.**

**A.** Representative images of TUNEL staining across different treatment groups (n = 5). Scale bars: 0.050 mm. **B.** Validation of c-Myc overexpression and knockdown efficiency using Western blot. **C.** Cell growth was assessed through a colony formation assay, with corresponding histogram quantification across various treatment groups. **D.** Transwell migration assays, along with histogram quantification, were performed to evaluate cell growth across different treatment groups. Data were mean ± SD. Statistical significance was calculated by 2-way ANOVA for **A**, **C** and **D**. ****P* < 0.001.


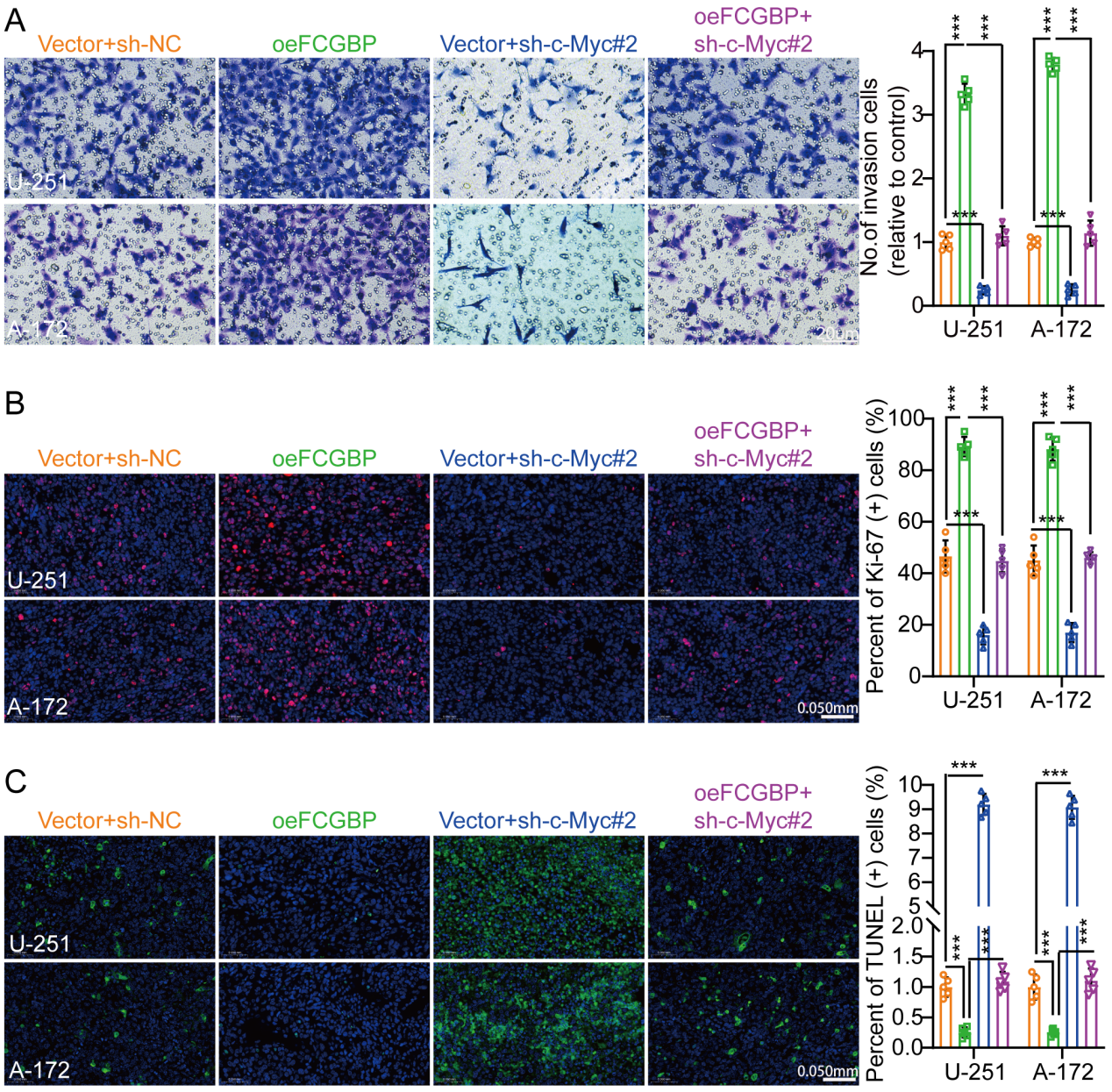


**Supplementary Figure 6: FCGBP promotes glioma progression through c-Myc.**

**A.** Transwell invasion assays, along with histogram quantification, were performed to assess cell growth across different treatment groups. **B-C.** Representative images of Ki-67 and TUNEL staining across various treatment groups (n = 5). Scale bars: 0.050 mm. Data were mean ± SD. Statistical significance was calculated by 2-way ANOVA for **A-C**. ****P* < 0.001.


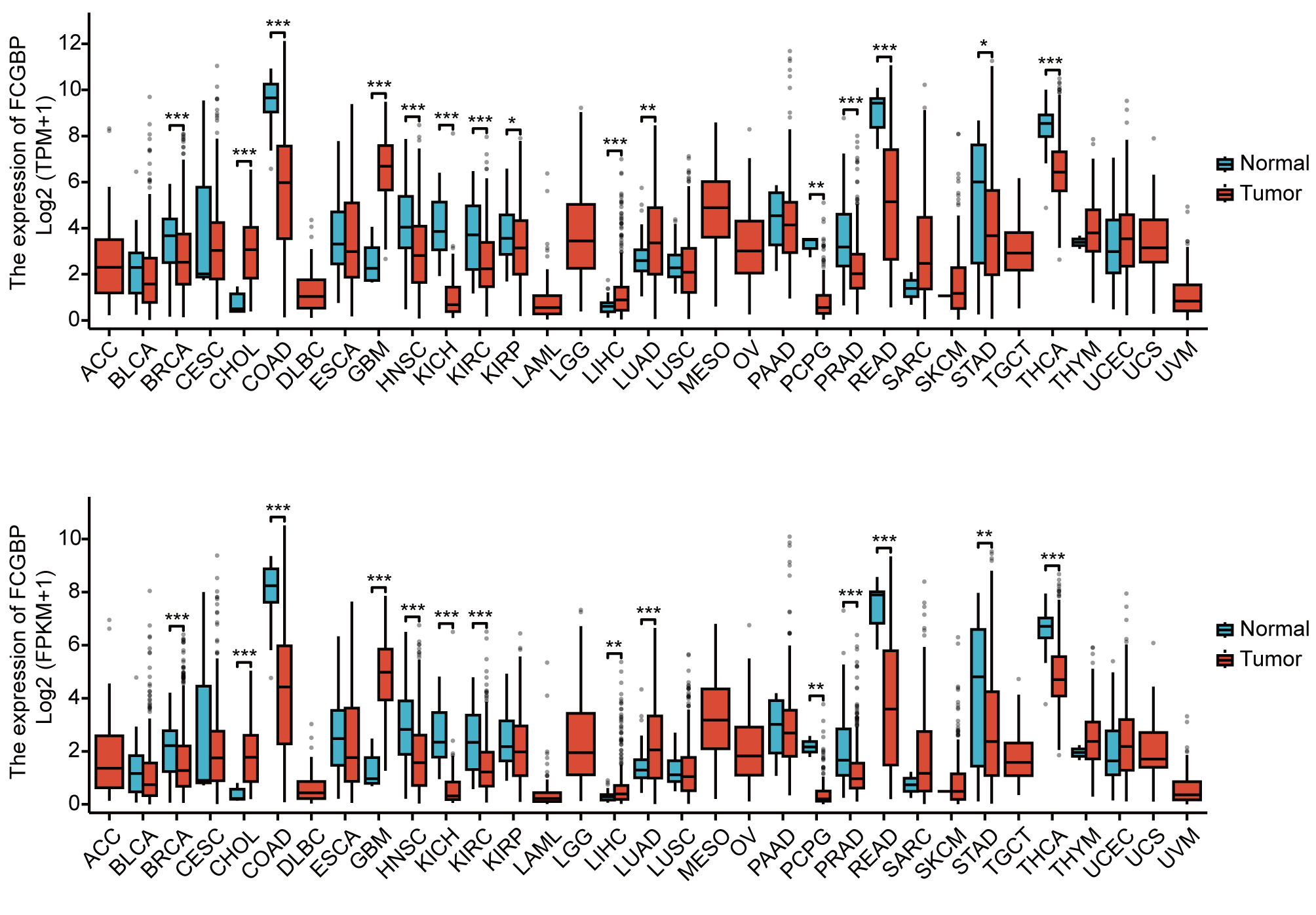


**Supplementary Figure 7: Pan-Cancer Database Analysis Results.** Pan-Cancer Database analysis also showed that FCGBP was significantly overexpressed in most tumors. Data were mean ± SD. Statistical significance was calculated by 2-tailed unpaired Student’s *t* tests. **P* < 0.01, ***P* < 0.01, ****P* < 0.001.


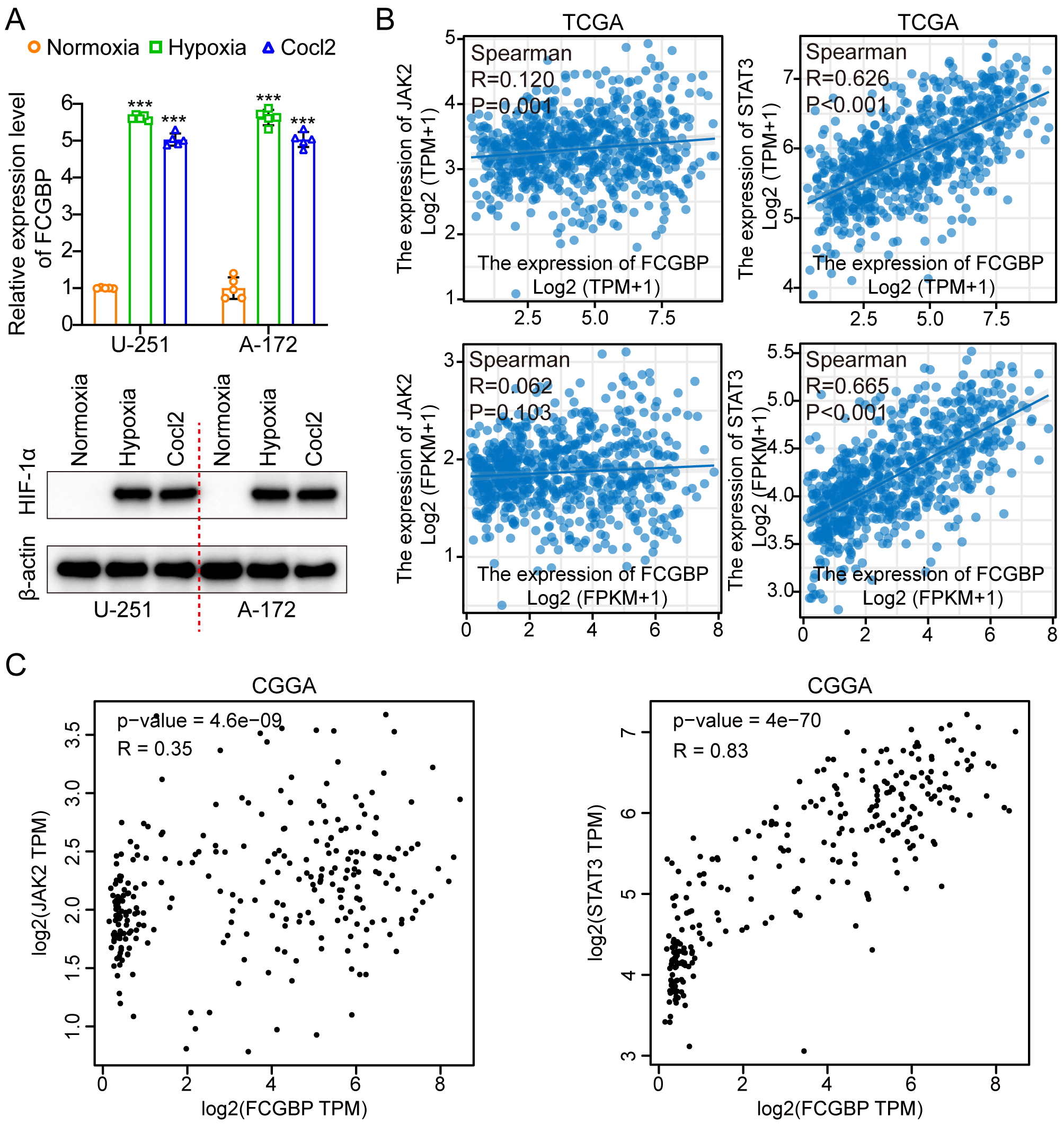


**Supplementary Figure 8:**

**A.** FCGBP expression levels under different treatment conditions. **B-C.** FCGBP expression is positively correlated with STAT3 signature scores across both TCGA and CGGA transcriptomic cohorts. Data were mean ± SD. Statistical significance was calculated by 1-way ANOVA for **A**; Spearman’s rank correlation test for **B** and **C**. ****P* < 0.001.


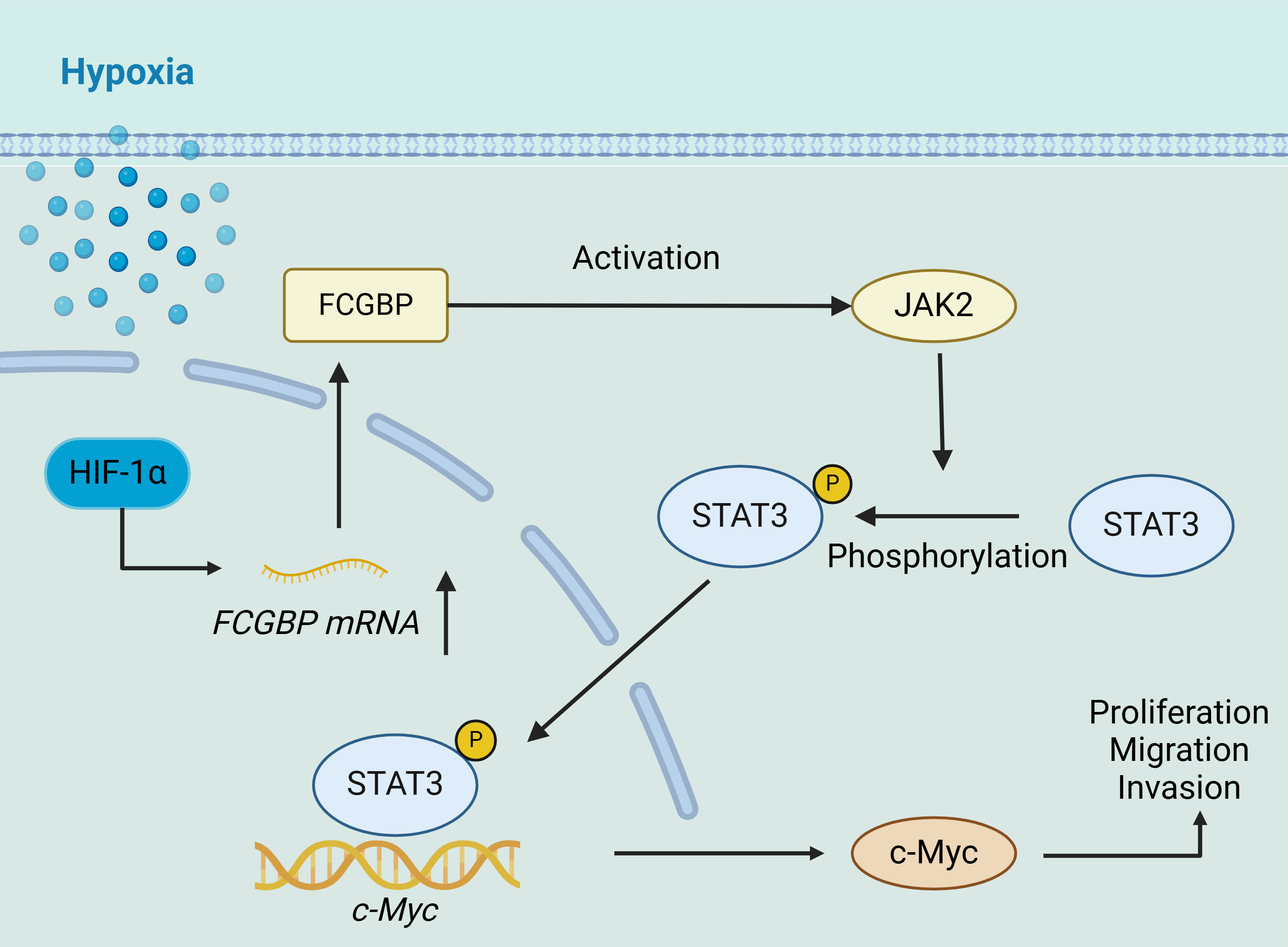


**Supplementary Figure 9: Schematic representation of the proposed mechanism.** FCGBP is highly expressed in gliomas and is negatively correlated with patient prognosis, contributing to glioma progression. Mechanistically, FCGBP promotes glioma progression through two key pathways: (1) FCGBP activates the JAK2-STAT3 signaling pathway, leading to the upregulation of c-Myc protein levels; (2) In the tumor's hypoxic microenvironment, HIF-1α directly binds to the FCGBP promoter, enhancing FCGBP protein expression.
